# Combined Rehabilitation with Alpha Lipoic Acid, Acetyl-L-Carnitine, Resveratrol, and Cholecalciferolin Discogenic Sciatica in Young People: A Randomized Clinical Trial

**DOI:** 10.3390/medicina59122197

**Published:** 2023-12-18

**Authors:** Dalila Scaturro, Fabio Vitagliani, Sofia Tomasello, Cristiano Sconza, Stefano Respizzi, Giulia Letizia Mauro

**Affiliations:** 1 Department of Surgical, Oncological and Stomatological Disciplines, University of Palermo, 90144 Palermo, Italy; giulia.letiziamauro@unipa.it; 2 Department of Biomedical and Biotechnological Sciences, University of Catania, 95123 Catania, Italy; fabiovitagliani93@libero.it; 3 Faculty of Medicine and Surgery, University of Palermo, 90127 Palermo, Italy; sofiatomasello@alice.it; 4 Department of Physical and Rehabilitation Medicine, Humanitas Clinical and Research Centre IRCSS, 20019 Milan, Italy; cristiano.sconza@humanitas.it (C.S.); stefano.respizzi@humanitas.it (S.R.)

**Keywords:** sciatica, rehabilitation, postural balance, alpha lipoic acid, acetyl-l-carnitine

## Abstract

*Background and Objectives*: In the Western world, back pain and sciatica are among the main causes of disability and absence from work with significant personal, social, and economic costs. This prospective observational study aims to evaluate the effectiveness of a rehabilitation program combined with the administration of Alpha Lipoic Acid, Acetyl-L-Carnitine, Resveratrol, and Cholecalciferol in the treatment of sciatica due to herniated discs in young patients in terms of pain resolution, postural alterations, taking painkillers, and quality of life. *Materials and Methods*: A prospective observational study was conducted on 128 patients with sciatica. We divided the sample into 3 groups: the Combo group, which received a combination of rehabilitation protocol and daily therapy with 600 mg Alpha Lipoic Acid, 1000 mg Acetyl-L-Carnitine, 50 mg Resveratrol, and 800 UI Cholecalciferol for 30 days; the Reha group, which received only a rehabilitation protocol; and the Supplement group, which received only oral supplementation with 600 mg Alpha Lipoic Acid, 1000 mg Acetyl-L-Carnitine, 50 mg Resveratrol, and 800 UI Cholecalciferol. Clinical assessments were made at the time of recruitment (T0), 30 days after the start of treatment (T1), and 60 days after the end of treatment (T2). The rating scales were as follows: the Numeric Rating Scale (NRS); the Oswestry Disability Questionnaire (ODQ); and the 36-item Short Form Health Survey (SF-36). All patients also underwent an instrumental stabilometric evaluation. *Results*: At T1, the Combo group showed statistically superior results compared to the other groups for pain (*p* < 0.05), disability (*p* < 0.05), and quality of life (*p* < 0.05). At T2, the Combo group showed statistically superior results compared to the other groups only for pain (*p* < 0.05) and quality of life (*p* < 0.05). From the analysis of the stabilometric evaluation data, we only observed a statistically significant improvement at T2 in the Combo group for the average X (*p* < 0.05) compared to the other groups. *Conclusions*: The combined treatment of rehabilitation and supplements with anti-inflammatory, pain-relieving, and antioxidant action is effective in the treatment of sciatica and can be useful in improving postural stability.

## 1. Introduction

The most common cause of low back pain (LBP) and sciatica is represented by lumbar disc herniation (LDH) [1].

About 9% of all people in the world are affected. It has a substantial impact on quality of life and represents a significant economic burden. In the Western world, back pain and sciatica are among the main causes of disability and absence from work with significant personal, social, and economic costs. In recent years, lifestyle changes have led to a gradual increase in the incidence of LDH and a reduction in the average age of onset [2].

Finally, it has been shown that people with back pain have worse physical and mental health than the healthy population. It has also been shown that there is a close relationship between pathology and psychosocial stress [3].

The approach to this pathology is multidisciplinary and includes medical therapy (NSAIDs, Glucocorticoids, Opioids, Muscle Relaxants, and Antiepileptics), interventional techniques such as intraforaminal injection of corticosteroids, physiotherapy in all its forms, and finally, surgery only for selected patients. The prolonged use of drugs or infiltrations is not recommended due to the numerous side effects or, in the second case, adverse events [4].

The updated 2017 LBP guidelines from the American College of Physicians recommend the use of nonpharmacologic treatments for patients with low back pain. Physical exercise is recommended in combination with other non-pharmacological therapies such as massage therapy and physical therapy [5].

Physiotherapy is considered the first-line treatment for patients with symptoms caused by a lumbar disc herniation, both in the acute and chronic phases. Some blind randomized controlled studies have demonstrated the effectiveness of the McKenzie method, mobilizations, and vertebral tractions, even in the acute phases [6].

Furthermore, some physical therapies such as cryotherapy, electrotherapy, laser therapy, etc., have been indicated as very sensitive initial accompanying treatments [7,8,9].

While in the chronic phases, the effectiveness of resistance exercises for muscle strengthening of the paravertebral and abdominal muscles has been demonstrated [10].

Some nutraceuticals are considered effective in neuroprotection and pain [1,2,3,4,5,6,7], although controlled studies in this regard are lacking. Alpha-lipoic acid (ALA), Acetyl-L-Carnitine (ALC), and palmitoylethanolamide (PEA) are effective in the treatment of neuropathic pain from root irradiation, as is Cholecalciferol, which has recognized analgesic properties [11,12,13,14,15].

This prospective observational study aimed to evaluate the effectiveness of a rehabilitation program combined with the administration of ALA, ALC, Resveratrol, and Cholecalciferol in the treatment of sciatica due to herniated discs in young patients in terms of pain resolution, postural alterations, taking painkillers, and quality of life.

## 2. Materials and Methods

### 2.1. Trial Design

We conducted a randomized controlled trial on outpatients who attended the U.O.C. outpatient clinics of Functional Recovery and Rehabilitation of the A.O.U. P. Paolo Giaccone of Palermo for lumbosciatica. The study period was between September 2022 and June 2023.

The study received approval from the local ethical committee “Palermo 1” (approval no. 08/2022) of the A.O.U.P. Paolo Giaccone of Palermo and was conducted in accordance with the declaration of Helsinki. The processing of information and data has been carried out according to the guidelines of Good Clinical Practice (GCP). Clinical trials registration number NCT06078163.

### 2.2. Participants

The inclusion criteria used were as follows: age 18–45 years; lower back pain with NRS scale score between 5 and 7 points; symptoms attributable to sciatica which occurred no more than 4 weeks ago; pharmacological washout of NSAIDs and/or corticosteroids for at least a week; lumbar MRI examination performed no more than 3 months ago; and written consent for participation in the study. Patients were excluded if they had altered states of consciousness; sciatic pain of non-disc origin; septic states in progress; presence of scoliosis >20° of Cobb; previous spinal surgery; or were pregnant and/or breastfeeding.

### 2.3. Intervention

The recruited patients were randomly divided into three groups through a system of computer-generated random numbers: the Combo group (CG), composed of patients subjected to a combination of a rehabilitation protocol of 20 sessions and daily therapy with 600 mg ALA, 1000 mg ALC, 50 mg Resveratrol, and 800 UI Cholecalciferol for 30 days; the Reha group (RG), made up of patients subjected only to a rehabilitation protocol lasting 20 sessions; and the Supplement group (SG), made up of patients who took 600 mg ALA, 1000 mg ALC, 50 mg Resveratrol, and 800 IU Cholecalciferol daily for 30 consecutive days.

All patients were asked to avoid taking NSAIDs during the study period but were given the option of 500 mg of Paracetamol in combination with 30 mg of Codeine as needed in case of excessive pain.

### 2.4. Outcomes

All recruited patients were evaluated three times: at the time of recruitment (T0), 30 days after the start of treatment (T1), and 60 days after the end of treatment (T2). During the initial clinical evaluation, demographic information (age, sex, BMI, education level) and clinical information (smoking habits and daily working hours) were collected. For each clinical evaluation, some rating scales were administered by the same physiatrist, such as the Numeric Rating Scale (NRS) [16], to evaluate the extent of the pain; the Oswestry Disability Questionnaire (ODQ) [17], to evaluate the degree of disability caused by low back pain; and the 36-item Short Form Health Survey (SF-36) questionnaire, to assess quality of life [18].

Patients were also asked to fill in a diary where they noted any daily intake of the combination of Paracetamol and Codeine and its frequency of intake during the day.

Finally, all patients will be subjected to stabilometric analysis using a baropodometric platform that uses the FreeMed posturography system (produced by Sensor Medica, Guidonia Montecelio, Roma, Italy). During the stabilometric examination, the length of the beam (mm) was considered, which is the size of the section drawn by the oscillation of the center of gravity (CoP) during the test; the surface of the ellipse (mm^2^), which includes 90% of the CoP section; the X-mean (mm), which indicates the average position maintained on the frontal plane during lateral oscillations; and the Y-mean (mm), which is the midpoint of the center of gravity on the sagittal plane during the anteroposterior oscillations [19].

### 2.5. Rehabilitation Protocol

The rehabilitation protocol to which the patients in the two groups (Combo and Reha) underwent was the same. It included daily sessions, 5 days a week, with a duration of 60 min, and for a total of 4 consecutive weeks. The rehabilitation protocol provided was carried out under the supervision of an experienced physiotherapist. We proceeded with an initial cardiorespiratory training phase lasting 15 min on a cycle ergometer (produced by Chinesport SPA, Udine, Italy). In the central part of the training, we proceeded with muscle strengthening exercises for the muscles of the trunk and the upper and lower part of the body and stretching exercises of the posterior kinetic chains. Each exercise was performed with 3 sets of 15 repetitions and at an intensity of 60% of the maximum. The third phase of the protocol involved therapy with physical agents, such as transcutaneous electronervous stimulation (TENS) for 20 min by stimulation with biphasic rectangular pulses of 100 microseconds and a frequency of 110 Hz, with a maximum output amplitude of 100 mA; and low power laser for 10 min with a stable, paravertebral, lumbar method with total daily doses of 18 J.

### 2.6. Rating Scales

The NRS scale is a quantitative rating scale by which patients are asked to rate their pain on a defined scale, from 0 to 10 [16].

The Oswestry Low Back Pain Disability Questionnaire (ODQ) (File S1) is a self-completed questionnaire with ten items covering pain intensity, ability to care for oneself, lifting and carrying, ability to walk, ability to sit, the ability to stand, the quality of sleep, social life, sexuality, and the ability to travel. Each item has six statements describing possible situations in the patient’s life. The most applicable statement is checked by the patient. Questions are scored on a scale of 0 to 5. We adapted the questionnaire by omitting an item regarding sexual function. The MCID for this scale is 10 points per second [17].

The SF-36 is a questionnaire comprising eight multiple-choice questions that can be divided into two large subgroups: the physical component of the disease and the mental component of the disease. A score is assigned to each scale; the higher the score, the better the state of mental and physical health. The score ranges from 0 (worst state of health) to 100 (best state of health). The MCID for this scale is 4.9 points [18].

### 2.7. Statistical Methods

The data collected were indexed in Excel. The aim was to detect a mean difference in NRS (0–10) between the two groups. A power analysis was conducted with the type I error set at 0.05 and the type II error at 0.15 (85% power). The estimated sample size was 45 patients from each group to detect the minimal clinically significant difference in NRS of 2.6 units [20]. The follow-up loss was estimated to be 20%. For this reason, the numbers of 44 patients for the Combo group, 43 patients for the Reha group, and 41 for the Supplement group were considered sufficient to demonstrate our thesis.

Through the use of the Shapiro–Wilk test, the normality of our collected data was verified. In the text and the tables, we have reported continuous variables, expressed as means and standard deviations, and categorical variables, expressed as absolute numbers and percentages.

Regarding the statistical analysis of the data, we used the *t*-test for the comparison of the means between the quantitative variables, while Mood’s median test was used for the comparison of the medians between the categorical variables. Finally, to evaluate the statistically significant difference between the NRS and lesion diameter variables examined between the two groups, ANOVA was used. R statistical software (R Core Team, Vienna, Austria, 2021) was used to analyze the collected data. A priori results showing *p* < 0.05 were considered statistically significant.

## 3. Results

Table 1 shows the general characteristics of the 128 patients who were included in the study. Participants were mainly women (59.8%), with an average age of 37.4 ± 3.4 years and an average BMI of 25.6 ± 2.8 kg/m^2^. A total of 48.3% (*n* = 42) had a primary school diploma, 32.2% (*n* = 28) had a secondary school diploma, and 19.5% (*n* = 17) had a university degree. More than half of the participants (63.2%) were smokers. The average daily working hours of the recruited sample were 9.6 ± 3.2 h. The mean perceived pain was 6.2 ± 0.6 points according to the NRS scale, with a mean score on the ODQ scale of 39.6 ± 5.1 and the SF-36 scale of 56.7 ± 7.2. There were no significant differences between the participants of the three study groups regarding the different baseline characteristics analyzed (Table 1).

Table 2 shows the changes in the variables examined in the three groups at T1. In the Combo group, we observed statistically significant improvements for perceived pain (6.4 ± 0.5 vs. 3.6 ± 0.3; *p* < 0.05) and for disability (40.2 ± 4.3 vs. 33.1 ± 3.7; *p* < 0.05). In the Reha group, statistically significant improvements were observed only for disability (38.8 ± 5.2 vs. 36.3 ± 4.2; *p* < 0.05). In the Supplement group, only pain showed statistically significant improvements after 30 days of treatment for pain reduction (6.4 ± 0.6 vs. 5.5 ± 0.5; *p* < 0.05).

Table 3 shows the changes in the variables examined in the three groups at T2. The Combo group showed statistically significant improvements for pain (6.4 ± 0.5 vs. 3.2 ± 0.4; *p* < 0.05), disability (40.2 ± 4.3 vs. 34.4 ± 4.2; *p* < 0.05), and quality of life (56.4 ± 5.8 vs. 81.6 ± 6.2; *p* < 0.05). The Reha group showed statistically significant improvements for pain (6.2 ± 0.7 vs. 4.1 ± 0.6; *p* < 0.05) and disability (38.8 ± 5.2 vs. 36.1 ± 3.9; *p* < 0.05). A statistically significant improvement was also observed in the Supplement group for pain (6.4 ± 0.6 vs. 4.8 ± 0.3; *p* < 0.05) and for disability (43.5 ± 3.2 vs. 37.2 ± 4.9; *p* < 0.05).

Table 4 shows the comparison between the results obtained in the three groups. At T1, the Combo group showed statistically superior results compared to the other groups regarding pain (*p* < 0.05), disability (*p* < 0.05), and quality of life (*p* < 0.05). At T2, the Combo group showed statistically superior results compared to the other groups only in terms of pain (*p* < 0.05) and quality of life (*p* < 0.05). No statistically significant differences were present between the three groups at T2 for disability.

Table 5 shows the results of the stabilometric examination in the three groups at T1 and T2. From the analysis of these data, we only observed a statistically significant improvement at T2 in the Combo group for the average X (*p* < 0.05) compared to the other groups.

Regarding the number of days of Paracetamol and Codeine intake, we observed a statistically significant reduction at T2 compared to T1 only in the treatment group (3.4 ± 0.8 vs. 1.8 ± 0.6; *p* < 0.05). At T2, however, no statistically significant difference was observed between the three groups.

## 4. Discussion

During this study, we tried to evaluate the effectiveness of the rehabilitation treatment combined with the administration of ALA, ALC, Resveratrol, and Cholecalciferol in the treatment of sciatica due to herniated discs in young patients. Efficacy was evaluated in terms of pain resolution, postural alterations, intake of painkillers, and quality of life.

From the results obtained, we observed how the rehabilitation treatment combined with the administration of supplements (ALA, ALC, Resveratrol, Cholecalciferol) showed statistically superior improvements in terms of reduction in pain and disability related to back pain, in the short term and above all in the long term, in the latter case also observing a significant improvement in terms of quality of life.

There are many non-pharmacological therapies for sciatic pain, and these include acupuncture, physical therapy, massage therapy, yoga, cognitive behavioral therapy or progressive relaxation, spinal manipulation, and intensive interdisciplinary rehabilitation. These treatments aim to control pain and above all to functionally recover patients, and although the level of evidence supporting the different therapies is discreet, at the moment, there is no consensus on their indication as first-choice treatments [21,22].

In addition to this, agents with antioxidant action, such as ALA and Resveratrol, have recently been identified as first-line treatment for chronic neuropathic pain [23,24,25] thanks to the proven efficacy compared to placebo in the treatment of neuropathic pain [26,27]. Oxidative stress that develops after peripheral neuropathic injury is considered a relevant factor responsible for neuropathic pain. It activates an inflammatory pathway that involves the entire peripheral nerve up to the spinal dorsal horn, causing sensitization and chronic neuropathic pain in the spinal column [28].

ALA can neutralize free radicals, but it also increases glutathione synthesis, regenerates other important antioxidants, and prevents the formation of glycosylated end products and mitochondrial damage from oxidative stress. Several studies have reported that ALA prevents oxidative damage to nerve tissue and nerve degeneration. Furthermore, ALA counteracts proinflammatory factors (including IL-6 and TNFα), thus reducing the overall inflammatory load. Indeed, recent reviews provide convincing evidence of the usefulness of ALA as an anti-inflammatory ally in several conditions such as acute and chronic pain, neuropathy, and ulcerative colitis [10].

Resveratrol is a widely used inhibitor of the WNT/β-catenin pathway, which plays a crucial role in many biological processes. It has shown anti-inflammatory effects in a rat arthritis model and also has well-described antioxidant properties [14].

ALC has important physiological and pharmacological actions due to its wide distribution in many tissues, including the brain [29]. Evidence from randomized controlled trials suggests that ALC is an effective agent for pain management in patients with peripheral neuropathies. The protective effects of ALC against nerve damage and pain associated with peripheral neuropathies include changes in the sensitivity of nerve growth factor (NGF) receptors, activation of M1 cholinergic muscarinic receptors in the CNS, and upregulation of glutamate receptors metabotropic type 2 (mGlu2) in dorsal root ganglion (DRG) neurons. More recently, activation of the phospholipase C (PLC)/inositol-1,4,4-triphosphate (PLC-IP 3) pathway and modulation of the transcriptional activity of nuclear factor (NF)-κB transcription factors through acetylation of the p65 subunit have been added to the list of potential mechanisms mediating the analgesic activity of ALC [13].

In line with our results, numerous studies have demonstrated how combinations of antioxidant (ALA and Resveratrol) and neurotrophic (LAC) agents can contribute to pain control, reducing the use of analgesic drugs and improving the safety profile of the treatments used [30,31,32,33].

An important observation from our research was the significant short-term reduction in the intake of painkillers. This is an important aspect to underline considering the multiple side effects resulting from their use. Among these, the most common are constipation, nausea, sedation, vomiting, and dizziness. Furthermore, with prolonged use of these drugs, many patients develop a physical and psychological dependence on opioids and the sudden cessation of the drug causes an unpleasant withdrawal syndrome (with agitation, insomnia, diarrhea, rhinorrhea, piloerection, and hyperalgesia) [34].

In the second part of our study, through stabilometric evaluation, we investigated any postural alterations that may be found in patients with sciatica. Recent studies indicate that patients with chronic low back pain have a decrease in postural control, manifesting balance problems. Postural balance is controlled by sensory information, central processing, and neuromuscular responses. Alterations in proprioception are identified as one of the possible causes of alterations in postural balance in subjects with low back pain. This type of pain is associated with decreased proprioception and muscle strength, which can affect the quality of information and compromise the relationship between postural responses and sensory responses to information [35].

Our data showed that the group subjected to the rehabilitation treatment combined with the administration of ALA, Resveratrol, LAC, and Cholecalciferol presented fewer perturbations in the frontal plane in the long term, without showing particularly significant improvements in all the other stabilometric parameters, both short and long term.

Pain represents the main factor responsible for changes in postural control, regardless of the intensity of the pain. This determines an alteration of the upright position which determines an increased activation of the lumbar muscles, with consequent increase in muscle fatigue [35,36,37].

In addition to pain, the impaired postural control found in patients with sciatic pain could result from a reduction in proprioceptive acuity, restrictive trunk movement, and protective trunk muscle strategies [38]. With the development of chronicity, a progressive decrease in variability and an increase in rigidity would lead to an increase in postural sway [38].

Several systematic reviews [35,38] suggest that low back pain leads to a shift in postural control from the lumbar spine to the ankles, and postural control at the ankles increases the magnitude of swing. However, other studies have reported smaller and slower CoP movements during quiet standing with eyes closed and eyes open [39].

Lemos et al. [40] analyzed the influence of low back pain on the balance of athletes of the Brazilian women’s canoe team and found an increase in the extent of displacement of the CoP in the horizontal plane in athletes with the presence of pain. Park et al. observed that, compared to controls, individuals with cLBP showed increased postural sway during quiet standing. In contrast, we did not find strong support for an effect of cLBP on postural swing velocity.

Our study was not without limitations. The first is represented by the small sample size which does not allow the results obtained to be generalized. Another limitation may be represented by the lack of evaluation between the different entities of lumbar pain and the postural alterations present in patients with sciatica. Finally, a further limitation may be represented by the failure to evaluate the stabilometric examination with eyes closed.

## 5. Conclusions

For the treatment of sciatica, the combined treatment with rehabilitation and supplements with anti-inflammatory, pain-relieving, and antioxidant action is effective in reducing pain and improving disability in the short term, compared to a single rehabilitation treatment or with supplements. In the long term, the combination also allows an improvement in the quality of life. Finally, the intake of natural substances reduces the need for painkillers.

## Figures and Tables

**Table 1:** General patient characteristics.

Characteristics	Total (*n* = 128)	Combo Group (*n* = 44)	Reha Group (*n* = 43)	Supplement Group (*n* = 41)	*p*-Value
Age, mean ± SD	37.4 ± 3.4	37.7 ± 2.8	37.2 ± 4.1	37.3 ± 3.1	0.69
Sex, no. (%)					
Male	50 (39.1)	16 (36.4)	19 (44.2)	15 (36.5)	0.71
Female	78 (60.9)	28 (63.6)	24 (55.8)	26 (63.5)	
BMI, mean ± SD	25.6 ± 2.8	25.8 ± 2.1	26.3 ± 2.2	25.6 ± 3.2	0.13
Education, no. (%)					
Primary school	60 (46.8)	22 (50)	20 (46.5)	18 (43.9)	0.56
Secondary school	38 (29.7)	15 (34.1)	13 (30.2)	10 (24.4)	
Degree	30 (23.5)	7 (15.9)	10 (23.3)	13 (31.7)	
Smoker, no. (%)					
Yes	84 (65.6)	26 (59.1)	29 (67.4)	29 (70.7)	0.43
No	44 (34.4)	18 (40.9)	14 (32.6)	12 (29.3)	
Work hours, mean ± SD	9.6 ± 3.2	9.9 ± 3.1	9.5 ± 2.9	9.7 ± 2.8	0.53
NRS, mean ± SD	6.2 ± 0.6	6.4 ± 0.5	6.2 ± 0.7	6.3 ± 0.6	0.13
ODQ, mean ± SD	39.6 ± 5.1	40.2 ± 4.3	38.8 ± 5.2	39.5 ± 4.9	0.7
SF-36, mean ± SD	56.7 ± 7.2	56.4 ± 5.8	57.8 ± 6.9	55.8 ± 7.7	0.31

**Table 2:** Effects of the different treatments in the Combo group, in the Reha group, and in the supplement group at T1.

Characteristics	Combo Group	Reha Group	Supplement Group
NRS, mean ± SD			
T0	6.4 ± 0.5	6.2 ± 0.7	6.3 ± 0.6
T1	3.6 ± 0.3	5.3 ± 0.5	5.5 ± 0.5
*p*-value	<0.05	0.13	<0.05
ODQ, mean ± SD			
T0	40.2 ± 4.3	38.8 ± 5.2	39.5 ± 4.9
T1	33.1 ± 3.7	36.3 ± 4.2	42.3 ± 4.2
*p*-value	<0.05	<0.05	0.81
SF-36, mean ± SD			
T0	56.4 ± 5.8	57.8 ± 6.9	55.8 ± 7.7
T1	64.6 ± 4.3	60.2 ± 7.2	58.2 ± 6.5
*p*-value	0.19	0.12	0.48

**Table 3:** Effects of the different treatments in the Combo group, in the Reha group, and in the supplement group at T2.

Characteristics	Combo Group	Reha Group	Supplement Group
NRS, mean ± SD			
T0	6.4 ± 0.5	6.2 ± 0.7	6.3 ± 0.6
T1	3.2 ± 0.4	4.1 ± 0.6	4.8 ± 0.3
*p*-value	<0.05	<0.05	<0.05
ODQ, mean ± SD			
T0	40.2 ± 4.3	38.8 ± 5.2	39.5 ± 4.9
T1	36.1 ± 3.9	36.1 ± 3.9	37.2 ± 4.9
*p*-value	<0.05	<0.05	<0.05
SF-36, mean ± SD			
T0	56.4 ± 5.8	57.8 ± 6.9	55.8 ± 7.7
T1	81.6 ± 6.2	61.5 ± 8.2	58.5 ± 7.2
*p*-value	<0.05	0.21	0.39

**Table 4:** Comparison of results at T1 and T2 between the three groups.

Characteristics	T1				T2			
	TG	CG	SG	*p*-Value	TG	CG	SG	*p*-Value
NRS, mean ± SD	3.6 ± 0.3	5.3 ± 0.5	5.5 ± 0.5	<0.05	3.2 ± 0.4	4.1 ± 0.6	4.8 ± 0.3	<0.05
ODQ, mean ± SD	33.1 ± 3.7	36.3 ± 4.2	42.3 ± 4.2	<0.05	34.4 ± 4.2	36.1 ± 3.9	37.2 ± 4.9	0.18
SF-36, mean ± SD	78.6 ± 4.3	60.2 ± 7.2	58.2 ± 6.5	<0.05	81.6 ± 6.2	61.5 ± 8.2	58.5 ± 7.2	<0.05

**Table 5:** Results of the stabilometric evaluation in the three groups.

Characteristics	T1				T2			
	CG	RG	SG	*p*-Value	CG	RG	SG	*p*-Value
Sphere length, mean ± SD	372.1 ± 124.8	374.3 ± 118.1	373.7 ± 114.5	0.93	366.2 ± 102.4	368.4 ± 105.2	368.6 ± 107.2	0.92
Ellipse surface, mean ± SD	131.1 ± 99.6	128.4 ± 98.7	129.5 ± 89.5	0.79	125.5 ± 92.5	124.6 ± 96.4	125.6 ± 94.5	0.96
Maximum oscillation, mean ± SD	2.5 ± 1.6	2.3 ± 1.5	2.3 ± 1.3	0.55	2.3 ± 1.1	2.2 ± 1.6	2.3 ± 1.4	0.73
X-media, mean ± SD	0.3 ± 7.1	0.5 ± 5.9	0.4 ± 6.3	0.98	0.2 ± 2.4	0.5 ± 5.2	0.4 ± 4.6	<0.05
Y-media, mean ± SD	−18.2 ± 11.9	−16.5 ± 10.6	−17.1 ± 10.2	0.48	−17.8 ± 10.6	−16.4 ± 10.4	−17.2 ± 9.8	0.53

## Data Availability

The data used to support the findings of this study are available from the corresponding author upon request.

## Supplementary Materials

The following supporting information can be downloaded at: https://www.mdpi.com/article/10.3390/medicina59122197/s1, File S1: The Oswestry Low Back Pain Disability Questionnaire (ODQ).
