# Supplementary material for: Combined Rehabilitation with Alpha Lipoic Acid, Acetyl-L-Carnitine, Resveratrol, and Cholecalciferolin Discogenic Sciatica in Young People: A Randomized Clinical Trial
Source: Medicina (Kaunas). 2023 Dec 18;59(12):2197. doi: 10.3390/medicina59122197 (PMC10744495; doi:10.3390/medicina59122197)
Supplement: Supplementary file 1 [file medicina-59-02197-s001.zip › medicina-2729630-supplementary.pdf]

OSWESTRY LOW BACK DISABILITY QUESTIONNAIRE

Instructions: this questionnaire has been designed to give us information as to how your back pain has affected your ability to manage everyday life. Please answer every section and mark in each section only the ONE box which applies to you at this time. We realize you may consider 2 of the statements in any section may relate to you, but please mark the box which most closely describes your current condition.

1. PAIN INTENSITY

- ◻ I can tolerate the pain I have without having to use pain killers
- ◻ The pain is bad but I manage without taking pain killers
- ◻ Pain killers give complete relief from pain
- ◻ Pain killers give moderate relief from pain
- ◻ Pain killers give very little relief from pain
- ◻ Pain killers have no effect on the pain and I do not use them

2. PERSONAL CARE (e.g. Washing, Dressing)

- ◻ I can look after myself normally without causing extra pain
- ◻ I can look after myself normally but it causes extra pain
- ◻ It is painful to look after myself and I am slow and careful
- ◻ I need some help but manage most of my personal care
- ◻ I need help every day in most aspects of self care
- ◻ I don't get dressed, I was with difficulty and stay in bed

3. LIFTING

- ◻ I can lift heavy weights without extra pain
- ◻ I can lift heavy weights but it gives extra pain
- ◻ Pain prevents me from lifting heavy weights off the floor, but I can manage if they are conveniently positioned, i.e. on a table
- ◻ Pain prevents me from lifting heavy weights, but I can manage light to medium weights if they are conveniently positioned
- ◻ I can lift very light weights
- ◻ I cannot lift or carry anything at all

4. WALKING

- ◻ Pain does not prevent me walking any distance
- ◻ Pain prevents me walking more than one mile
- ◻ Pain prevents me walking more than ½ mile
- ◻ Pain prevents me walking more than ¼ mile
- ◻ I can only walk using a stick or crutches
- ◻ I am in bed most of the time and have to crawl to the toilet

5. SITTING

- ◻ I can sit in any chair as long as I like
- ◻ I can only sit in my favorite chair as long as I like
- ◻ Pain prevents me from sitting more than one hour
- ◻ Pain prevents me from sitting more than ½ hour
- ◻ Pain prevents me from sitting more than 10 minutes
- ◻ Pain prevents me from sitting at all

6. STANDING

- ◻ I can stand as long as I want without extra pain
- ◻ I can stand as long as I want but it gives me extra pain
- ◻ Pain prevents me from standing for more than one hour
- ◻ Pain prevents me from standing for more than 30 minutes
- ◻ Pain prevents me from standing for more than 10 minutes
- ◻ Pain prevents me from standing at all

7. SLEEPING

- ◻ Pain does not prevent me from sleeping well
- ◻ I can sleep well only by using medication
- ◻ Even when I take medication, I have less than 6 hrs sleep
- ◻ Even when I take medication, I have less than 4 hrs sleep
- ◻ Even when I take medication, I have less than 2 hrs sleep
- ◻ Pain prevents me from sleeping at all

8. SOCIAL LIFE

- ◻ My social life is normal and gives me no extra pain
- ◻ My social life is normal but increases the degree of pain
- ◻ Pain has no significant effect on my social life apart from limiting my more energetic interests, i.e. dancing, etc.
- ◻ Pain has restricted my social life and I do not go out as often
- ◻ Pain has restricted my social life to my home
- ◻ I have no social life because of pain

9. TRAVELLING

- ◻ I can travel anywhere without extra pain
- ◻ I can travel anywhere but it gives me extra pain
- ◻ Pain is bad, but I manage journeys over 2 hours
- ◻ Pain restricts me to journeys of less than 1 hour
- ◻ Pain restricts me to short necessary journeys under 30 minutes
- ◻ Pain prevents me from traveling except to the doctor or hospital

10. EMPLOYMENT/ HOMEMAKING

- ◻ My normal homemaking/ job activities do not cause pain.
- ◻ My normal homemaking/ job activities increase my pain, but I can still perform all that is required of me.
- ◻ I can perform most of my homemaking/ job duties, but pain prevents me from performing more physically stressful activities (e.g. lifting, vacuuming)
- ◻ Pain prevents me from doing anything but light duties.
- ◻ Pain prevents me from doing even light duties.
- ◻ Pain prevents me from performing any job or homemaking chores

SF-36 QUESTIONNAIRE

Name:____________________ Date:___________

ID#: _______________ Gender: M / F

Please answer the 36 questions of the **Health Survey** completely, honestly, and without interruptions.

GENERAL HEALTH:
In general, would you say your health is:

- Excellent
- Very Good
- Good
- Fair
- Poor

Compared to one year ago, how would you rate your health in general now?

- Much better now than one year ago
- Somewhat better now than one year ago
- About the same
- Somewhat worse now than one year ago
- Much worse than one year ago

LIMITATIONS OF ACTIVITIES:
The following items are about activities you might do during a typical day. Does your health now limit you in these activities? If so, how much?

Vigorous activities, such as running, lifting heavy objects, participating in strenuous sports.
- Yes, Limited a lot
- Yes, Limited a Little
- No, Not Limited at all

Moderate activities, such as moving a table, pushing a vacuum cleaner, bowling, or playing golf
- Yes, Limited a Lot
- Yes, Limited a Little
- No, Not Limited at all

Lifting or carrying groceries
- Yes, Limited a Lot
- Yes, Limited a Little
- No, Not Limited at all

Climbing several flights of stairs
- Yes, Limited a Lot
- Yes, Limited a Little
- No, Not Limited at all

Climbing one flight of stairs
- Yes, Limited a Lot
- Yes, Limited a Little
- No, Not Limited at all

Bending, kneeling, or stooping
- Yes, Limited a Lot
- Yes, Limited a Little
- No, Not Limited at all

Walking more than a mile
- Yes, Limited a Lot
- Yes, Limited a Little
- No, Not Limited at all

Walking several blocks
- Yes, Limited a Lot
- Yes, Limited a Little
- No, Not Limited at all

Walking one block
- Yes, Limited a Lot
- Yes, Limited a Little
- No, Not Limited at all

Bathing or dressing yourself

◯ Yes, Limited a Lot ◯ Yes, Limited a Little ◯ No, Not Limited at all

PHYSICAL HEALTH PROBLEMS:

During the past 4 weeks, have you had any of the following problems with your work or other regular daily activities as a result of your physical health?

Cut down the amount of time you spent on work or other activities

◯ Yes ◯ No

Accomplished less than you would like

◯ Yes ◯ No

Were limited in the kind of work or other activities

◯ Yes ◯ No

Had difficulty performing the work or other activities (for example, it took extra effort)

◯ Yes ◯ No

EMOTIONAL HEALTH PROBLEMS:

During the past 4 weeks, have you had any of the following problems with your work or other regular daily activities as a result of any emotional problems (such as feeling depressed or anxious)?

Cut down the amount of time you spent on work or other activities

◯ Yes ◯ No

Accomplished less than you would like

◯ Yes ◯ No

Didn't do work or other activities as carefully as usual

◯ Yes ◯ No

SOCIAL ACTIVITIES:

Emotional problems interfered with your normal social activities with family, friends, neighbors, or groups?

◯ Not at all ◯ Slightly ◯ Moderately ◯ Severe ◯ Very Severe

PAIN:

How much bodily pain have you had during the past 4 weeks?

◯ None ◯ Very Mild ◯ Mild ◯ Moderate ◯ Severe ◯ Very Severe

During the past 4 weeks, how much did pain interfere with your normal work (including both work outside the home and housework)?

◯ Not at all ◯ A little bit ◯ Moderately ◯ Quite a bit ◯ Extremely

ENERGY AND EMOTIONS:
These questions are about how you feel and how things have been with you during the last 4 weeks. For each question, please give the answer that comes closest to the way you have been feeling.

Did you feel full of pep?
- All of the time
- Most of the time
- A good Bit of the Time
- Some of the time
- A little bit of the time
- None of the Time

Have you been a very nervous person?
- All of the time
- Most of the time
- A good Bit of the Time
- Some of the time
- A little bit of the time
- None of the Time

Have you felt so down in the dumps that nothing could cheer you up?
- All of the time
- Most of the time
- A good Bit of the Time
- Some of the time
- A little bit of the time
- None of the Time

Have you felt calm and peaceful?
- All of the time
- Most of the time
- A good Bit of the Time
- Some of the time
- A little bit of the time
- None of the Time

Did you have a lot of energy?
- All of the time
- Most of the time
- A good Bit of the Time
- Some of the time
- A little bit of the time
- None of the Time

Have you felt downhearted and blue?

- All of the time
- Most of the time
- A good Bit of the Time
- Some of the time
- A little bit of the time
- None of the Time

Did you feel worn out?

- All of the time
- Most of the time
- A good Bit of the Time
- Some of the time
- A little bit of the time
- None of the Time

Have you been a happy person?

- All of the time
- Most of the time
- A good Bit of the Time
- Some of the time
- A little bit of the time
- None of the Time

Did you feel tired?

- All of the time
- Most of the time
- A good Bit of the Time
- Some of the time
- A little bit of the time
- None of the Time

SOCIAL ACTIVITIES:
During the past 4 weeks, how much of the time has your physical health or emotional problems interfered with your social activities (like visiting with friends, relatives, etc.)?

- All of the time
- Most of the time
- Some of the time
- A little bit of the time
- None of the Time

GENERAL HEALTH:
How true or false is each of the following statements for you?

I seem to get sick a little easier than other people
Definitely true Mostly true Don't know Mostly false Definitely false

I am as healthy as anybody I know
Definitely true Mostly true Don't know Mostly false Definitely false

I expect my health to get worse
Definitely true Mostly true Don't know Mostly false Definitely false

My health is excellent
Definitely true Mostly true Don't know Mostly false Definitely false
